# Piezoelectric Effect of Antibacterial Biomimetic Hydrogel Promotes Osteochondral Defect Repair

**DOI:** 10.3390/biomedicines10051165

**Published:** 2022-05-18

**Authors:** Jiahang Wu, Taijun Chen, Yingying Wang, Jiafan Bai, Chenwen Lao, Minyue Luo, Mingxia Chen, Wenzhen Peng, Wei Zhi, Jie Weng, Jianxin Wang

**Affiliations:** 1 Key Laboratory of Advanced Technologies of Materials, Ministry of Education, School of Materials Science and Engineering, Southwest Jiaotong University, Chengdu 610031, China; 19923809565@163.com (J.W.); tychentaijun@163.com (T.C.); yingying1045@163.com (Y.W.); baijiaf2009@126.com (J.B.); laochengwen@126.com (C.L.); luominyue3826@163.com (M.L.); chenmingxia1205@163.com (M.C.); zhiwei@home.swjtu.edu.cn (W.Z.); jweng@swjtu.edu.cn (J.W.); 2 Chengdu Yikeda Biotechnology Co., Ltd., Chengdu 610095, China; halishengwu@163.com

**Keywords:** piezoelectric effect, biomimetic hydrogel, antibacterial, cartilage tissue, tissue regeneration

## Abstract

The lack of vascular tissue and the low metabolism and biological activity of mature chondrocytes lead to the low regeneration ability of articular cartilage. People try to solve this problem through various methods, but the effect is not very ideal. Inspired by the piezoelectric effect of collagen in cartilage tissue, this work focused on the design of a biomimetic hydrogel by introducing piezoelectric materials and silver nanowires into hydrogel to endow them with piezoelectric and antibacterial properties to promote tissue regeneration. Additionally, the mechanical and swelling properties of the material were adjusted to match natural articular cartilage. Based on bionic principles, a double-layer piezoelectric hydrogel was prepared and applied for the repair of osteochondral defects. An enhanced repair effect of osteochondral defects has been seen, which has demonstrated potential values for future application in bionics principle- and piezoelectric effect-based osteochondral tissue engineering. Furthermore, piezoelectric effect-induced degradation was observed. These results fully indicated the positive effect of the piezoelectric effect on promoting the regeneration of osteochondral tissue and in vivo degradation of materials.

## 1. Introduction

Articular cartilage is one of the connective tissues in the skeletal system. However, due to the fact that cartilage is an avascular tissue, the metabolism and biological activity of mature chondrocytes are low, resulting in a low regeneration ability. Once damaged, it leads to long-term functional impairment [1].

Although the known surgical treatment for cartilage disease has a certain effect, it cannot completely repair the damaged defect. Therefore, to improve the quality of treatment, it is necessary to seek other methods to solve this problem. In general, articular cartilage has a structure similar to that of hydrogels [2,3]. Therefore, the application of hydrogels in repairing articular cartilage injury has attracted increasing attention.

Because of its good biocompatibility and low immune response, hydrogels are a promising biomaterial for osteochondral tissue engineering. Pulkkinen et al. demonstrated that type II collagen hydrogels were beneficial for the proliferation and chondrogenesis of mesenchymal stem cells [4]. Park et al. reported an injectable, click-crosslinked, cytomodulin-modified hyaluronic acid hydrogel and applied it for cartilage tissue engineering [5]. The increased expression of type II collagen, glycosaminoglycan content and SOX9, aggrecan, and type 2 α 1 collagen mRNA levels were observed. Zhu et al. constructed a bilayer hydrogel scaffold based on bacterial cellulose-enhanced double-network hydrogel. Without using any growth factor, both good integration between the neo-subchondral bone and the surrounding host bone and the same thickness of the new cartilage as that of the host cartilage were realized at 12 weeks postoperatively [3]. However, traditional hydrogels lack the function of initiating activating cell regeneration in repairing tissue. Currently, the methods of regulating cell regeneration activity by physical stimulations have been proved to be feasible [6] (such as pH stimulation, light stimulation, and electrical stimulation). Rahimi et al. designed an electrically responsive hydrogel composed of polyacrylic acid (PAAC) and fibrin [7]. Under the electric field, it can guide the arrangement of muscle cells and promote the distribution of cells in the structure. Xiao et al. used self-assembled acrylate block copolymer micelles to prepare mechanical responsive hydrogels [8]. The results showed that the elastic hydrogel could effectively control drug release through reversible deformation and tissue repair. However, there are many problems in the use of external electric fields, such as expensive equipment, inconvenient operation, and so on. 

Inspired by the piezoelectric effect of collagen in cartilage tissue [9,10], this work would focus on the design of a biomimetic hydrogel by introducing piezoelectric materials into hydrogel to endow hydrogels with piezoelectric properties, thereby generating electrical signals under the stress field of hydrogels to stimulate cell proliferation and differentiation, and promote tissue regeneration. Feng et al. first reported on the construction of a piezoelectric hydroxyapatite and barium titanate bioceramic [11]. Their results showed that such piezoelectric bioceramic could greatly improve the regeneration and repair of the bone. Ma et al. demonstrated the regulatory effect of piezoelectric nylon-11 nanoparticles on stem cell differentiation [12]. However, to date, there has not been any report on the application of the piezoelectric effect in osteochondral repair. Whether there is a similar mechanism of piezoelectric-enhanced osteochondral regeneration remains unknown. To verify this, our strategy was to hope that the material itself has a certain repair performance, but it is not so prominent. If the repair effect of the material itself is too good, it will mask the repair effect brought by the piezoelectric effect. Therefore, we would choose the traditional polyvinyl alcohol (PVA) hydrogel as the osteochondral repair material. 

Although PVA has been proven to be able to be used in the repair of osteochondral defects in previous work, due to its low biodegradability and bioactivity only pure PVA hydrogel cannot obtain good osteochondral repair ability [13,14]. However, these results will help us to intensively study the effect of piezoelectric performance on osteochondral repair and its mechanism of promoting cartilage formation in such PVA systems. In this study, we would design the polyvinyl alcohol (PVA)/ polyvinylidene fluoride (PVDF) composite hydrogel into a double-layer structure to imitate the osteochondral tissue structure. Silver nanowires would be added into the cartilage layer to induce the formation of β-PVDF phase (piezoelectric phase) to improve the piezoelectric properties of PVDF and to endow hydrogel with certain antibacterial properties. In addition, nano hydroxyapatite (nano-HA) was added to the bone layer to improve the compressive modulus and osteogenic ability of the material [3]. Finally, the osteochondral repair effect and piezoelectric enhancement mechanism were evaluated using the rabbit osteochondral defect model.

## 2. Materials and Methods

### 2.1. Materials

Sodium chloride (NaCl, >98.0%), Dimethyl sulfoxide (DMSO, >98.0%), Silver nitrate (AgNO_3_, 99%), Absolute ethanol (99%), Calcium nitrate tetrahydrate (Ca(NO_3_)_2_·4H_2_O), Ammonium phosphate ((NH_4_)_2_HPO_4_), Ammonia, Polyvinylpyrrolidone (PVP, K30), Trypsin and Poly(vinyl-alcohol) (PVA, 1799) were purchased from Kelong Chemical Reagent Co., Ltd. (Chengdu, China). Poly(vinylidene fluoride) (PVDF) (Mw = 275,000) was obtained from Shanghai Maichao Co., Ltd. (Shanghai, China). Other reagents used in this work were of analytical grade.

### 2.2. Preparation of Hydrogels

PVA/PVDF piezoelectric hydrogel was prepared using a two-step method. First, 60 mL DMSO and 40 mL distilled water were mixed into a solvent, and 7.5 g PVA and 7.5 g PVDF were dissolved in this solvent. The mixture was stirred for 6 h at 92 °C until the suspension solution became homogeneous. Subsequently, the solution was poured into the mold, frozen, and thawed for three cycles [15]. Finally, the PVA/PVDF piezoelectric hydrogel was obtained and dehydrated with gradient alcohol.

### 2.3. Preparation of Nano-HA

The synthesis of nano-HA was based on the method reported previously [16]. 11.8 g calcium nitrate tetrahydrate (Ca(NO_3_)_2_·4H_2_O) was dissolved in 100 mL DD water, and 3.95 g ammonium phosphate ((NH_4_)_2_HPO_4_) were dissolved in 60 mL DD water. The (NH_4_)_2_HPO_4_ was added into the Ca(NO_3_)_2_·4H_2_O to obtain a mixed solution under stirring, and the mixed solution was adjusted to pH = 11 using ammonia. Subsequently, ethylenediamine tetraacetic acid (7.3 g) was added to the mixed solution, the final mixture was transferred into a Teflon-lined autoclave, and the hydrothermal reaction was carried out at 180 °C for 6 h. The products were washed with deionized water under vacuum filtration, then dried at 80 °C and sintered at 900 °C to obtain nano-HA.

### 2.4. Preparation of Silver Nanowires

The preparation method of silver nanowires (Ag-NWs) is based on the ethylene glycol system reported previously [17], and the specific operation process is as follows: 70 mL PVP (K30) glycol solution with 1.43 *w/v* % was prepared and heated to 180 °C. After 5 min, the same volume and concentration of NaCl and AgNO_3_ solution (45 μL, 0.431 mol/L) were added to the above system to form AgCl seed colloid. After stirring for 5–10 min, 25 mL AgNO_3_ solution with a concentration of 0.174 mol/L were slowly added. After the addition of AgNO_3_ solution, the reaction was continued for 5 min, and the reaction was finished. Subsequently, silver nanowires were obtained by repeated centrifugal rinsing with ethanol.

### 2.5. Preparation of Bi-Layer Hydrogel

The preparation process of the bi-layer hydrogel is as follows. The preparation method of osteogenic layer and chondrogenic layer is similar to that of single layer hydrogel; 0.6 g of Ag-NWs was added into the solution system as chondrogenic layer, while 7 g of nano-HA was added into the solution system as osteogenic layer. Subsequently, the solution of the osteogenic layer was poured into the mold, and then the composition for the chondrogenic layer was also poured into the same mold. After these steps were completed, the mixture was quickly placed in the refrigerator to freeze and shape. Finally, the double-layer piezoelectric gel was obtained by freezing—thawing three times. 

### 2.6. Characterization of Materials

The composite mechanism of the sample was analyzed using a Fourier transform infrared spectrometer (FT-IR, PerkinElmer, Waltham, MA, USA). The compressive stress of the hydrogels was measured by a universal tensile machine INSTRON-5967. The dynamic mechanics of the materials were used to test by a DHR-3 rheometer (TA Instruments, New Castle, DE, USA). Piezoelectric properties of the materials were tested using a digital source meter (keithley6514). The morphology of the hydrogels was observed at 15 kV accelerating voltage using scanning electron microscopy (SEM, Quanta 200, Philips, Eindhoven, Netherlands). The samples were lyophilized and then sputter coated with gold before SEM observation and scanning electron microscopy energy dispersive X-ray spectroscopy (SEM-EDS) analysis. The swelling properties of the samples were measured using the weighing method. The as-prepared hydrogels were immersed in phosphate-buffered saline (PBS) until swelling equilibrium was attained. The swelling ratio of the gels was calculated from the following equation: SR = (Ws − Wd)/ Wd × 100%, where SR is the swelling ratio, Ws is the mass of swollen hydrogel, and Wd is the mass of dried hydrogel.

### 2.7. Cytocompatibility

The cytocompatibility of the hydrogel samples was evaluated through the co-culture of L929 cells with PVA/HA hydrogel and PVA/PVDF/Ag/HA hydrogel. These L929 cells co-cultured with PBS were set as the blank control group (*n* = 6). The cell proliferation was detected by using a UV-VIS spectrometer at a wavelength of 570 nm at days 1, 3, 5, and 7 of co-culture, respectively, by using MTT assay, and the cell morphology was observed and photographed by using a fluorescence microscope (Olympus, Tokyo, Japan) after staining with AO/PI fluorescent dye at day 7 of co-culture.

### 2.8. Hemolysis Assay

The hydrogel samples with a diameter of 5 mm and a height of 2~3 mm were prepared. The rabbit blood was centrifuged at 1500 rpm for 15 min at 4 °C, rinsed with PBS, and the cycle was repeated three times. The erythrocytes were diluted in PBS to obtain a 2% (*v*/*v*) erythrocyte suspension. 1 mL erythrocyte suspension was mixed with 4 mL of PBS, deionized water served as the negative and positive controls, respectively, and hydrogels were put into PBS/erythrocyte suspension solution as the experimental groups. All the mixtures were incubated at 37 °C for 2 h. After the samples were removed, the mixtures were centrifuged at 5000 r/min for 5 min. 200 μL of the supernatants were transferred into a new 96-well plate, and OD540 was measured using a microplate reader. The hemolysis rate was calculated using the formula HP = (Dt − Dnc)/(Dpc − Dnc) × 100%, where HP, Dt, Dnc, and Dpc were the hemolysis rate and the absorbance values of the experimental group, the negative control group, and the positive control group, respectively.

### 2.9. Degradation Experiment of Hydrogel

The degradation test was performed in 30 mL PBS (pH = 7.4) or PBS/trypsin solu-tion. The weighed samples were immersed in a certain medium and incubated in a thermostatic bath at 37 °C. At predetermined time points, the samples were removed from the medium and then lyophilized. The degradation rate was assessed by measuring the weight ratio (%), which was defined with the following formula: Degradation rate (%) = (W0 − W1)/W0 × 100%, here W0 and W1 are the weight of the initial gels and after degradation, respectively.

### 2.10. Antibacterial Test

Gram-positive *S. aureus* and Gram-negative *E. coli* were used to evaluate the antibacterial properties of the hydrogel samples. The hydrogel samples with 3~4 mm height, 10 mm diameter were used as the experimental group, while clinical gauze was used as the control group. The bacterial suspension was diluted with beef paste agar liquid medium to form a bacterial suspension with the final concentration of 10^6^ CFU/mL. 100 µL of such bacterial suspension and quantitative sterile liquid medium were added to immerse the sample in the test tube. After incubation at 37 °C for 24 h, 50 μL of bacterial suspension was evenly smeared on the beef paste agar plate with a scraper. To quantify the antibacterial property, the density of bacteria was detected by the colony counting method, and the antibacterial rate (AR) was calculated using the formula AR = (Ncontrol − Nsample)/Ncontrol × 100%, where Ncontrol and Nsample were the average number of bacteria colonies of the control group and the sample group, respectively.

### 2.11. Animal Model and Histological Analysis

All surgical procedures and subsequent animal care were approved by Ethics Committee of State Key Laboratory of Oral Diseases, West China Hospital of Stomatology (Approval Code: SKLODLL2013A86; Approval Date: 25 February 2013). A total of 30 female New Zealand White rabbits of 6 weeks old were used in this study and randomly divided into three groups, namely, the blank group (nonmaterial implanted), PVA/HA group and PVA/PVDF/Ag/HA group (*n* = 10 for each group). The animals were anesthetized with an injection of 10% (*w*/*v*) chloral hydrate (2 mL/kg) into the ear-vein. The right knee of the rabbit was shaved, sterilized using iodine solution, and draped in a sterile manner, followed by an arthrotomy of the knee joint performed by an anterior medial parapatellar incision, and an operation of everting the patella. The full thickness osteochondral defects (φ5 × H5 mm) were created on the trochlear groove using a surgical drill, and then the joint was irrigated immediately with sterile isotonic saline. After removing the debris from the defect with a curette, the defect edge was trimmed and cleaned with a scalpel blade. The samples were implanted into the defects. The wounds were then carefully sewed up with a standard surgical procedure. After surgery, the animals were administered penicillin (100,000 U/kg) once each day for three consecutive days. The animals were monitored every day post-surgery. Rabbits were sacrificed at the end of 12 weeks, and the joint specimens of the distal femurs were collected and fixed in 10% buffered formalin. After we harvested those joint specimens, the macroscopic observation results were obtained by two observers based on the International Cartilage Repair Society (ICRS) scores system (Table 1) and Histological scoring system (Table 2). After decalcification in 10% EDTA solution, the specimens were cut into sections of 5 µm thickness.

The H&E staining procedure is briefly described as follows: The tissue sections were incubated in the hematoxylin solution at 37 °C for 5 min, rinsed with water for 1 min to remove excess hematoxylin, and then incubated in 1% hydrochloric acid and ethanol for 30 s and rinsed with water for 1 min. After rinsing, they were incubated in 0.5% eosin solution for 3 min and re-rinsed with distilled water for 1 min.

The toluidine blue staining procedure is briefly described as follows: The tissue sections were incubated in 0.2% toluidine blue solution for 10 min and rinsed with water for 3 s.

### 2.12. Statistical Analysis

All data are presented as mean ± standard deviation. Student’s test was used to compare the data between two groups, and one-way analysis of variance (ANOVA) was used for statistical tests for comparison between multiple groups. If the p-value is lower than 0.05, the difference was considered statistically significant. The displayed error bars represent the standard deviation (SD).

## 3. Results and Discussion

### 3.1. Optimization of PVA/PVDF Hydrogels 

For materials used to repair osteochondral defects, we hope that they have certain piezoelectric properties to stimulate the activity of the cells, and that they also have certain mechanical properties and appropriate swelling rates to adapt to the physiological environment of cartilage. Therefore, we first conducted some tests on swelling and piezoelectric and mechanical properties. Based on these results, we screened out the most suitable PVA/PVDF materials for the above purposes.

**Figure 1:**
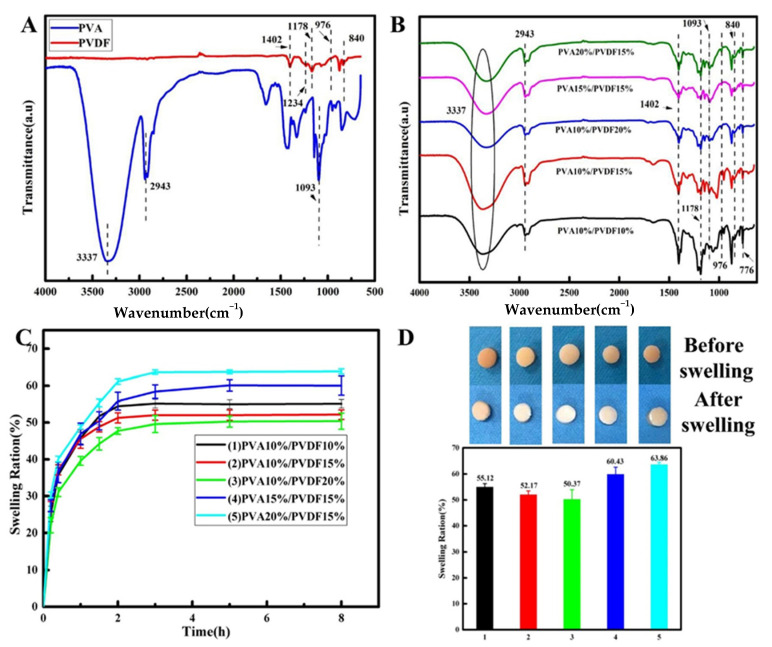
FT-IR analysis and swelling behaviors of the hydrogels. (**A**) FI-IR spectra of pure PVA and PVDF. (**B**) FT-IR spectra of PVA/PVDF composite hydrogels. (**C**) Swelling curves of hydrogels in PBS buffer solution at 37 °C, pH = 7.4. (**D**) Swelling ratio of hydrogels and images of the hydrogels before and after swelling equilibrium.

Figure 1A. shows the FT-IR characteristic peaks of PVA and PVDF. For PVA, the peaks at 3337 cm^−1^, 2943 cm^−1^ and 1093 cm^−1^ corresponded to the stretching vibration peak of -OH [18], the asymmetrical stretching vibration peak of -CH_2_ and the stretching vibration peak of C-O, respectively. For pure PVDF, the peaks at 1402 cm^−1^ and 1178 cm^−1^ were attributed to the stretching vibration peaks of -CH_2_ and C-F. The piezoelectric property of PVDF is mainly reflected in the characteristic peak of its piezoelectric β-phase at 840 cm^−1^. For this reason, the FT-IR spectrum of PVA/PVDF composites was investigated, as shown in Figure 1B. The characteristic peaks of α-phase, γ-phase and β-phase appeared at 976 cm^−1^, 1234 cm^−1^ and 840 cm^−1^, respectively [19,20]. Compared with the spectra of pure PVA and pure PVDF, the characteristic peaks of PVA/PVDF composites showed no obvious shift or change. The results indicated that no chemical bonds were formed between PVA and PVDF, and the characteristic peak of the β-phase at 840 cm^−1^ still existed; therefore, we inferred that the as-obtained composite materials should have the piezoelectric effect. In order to prove this conjecture, the piezoelectric property of the composites was tested, and the effects of PVA and PVDF content on the piezoelectric property were also investigated.

**Figure 2:**
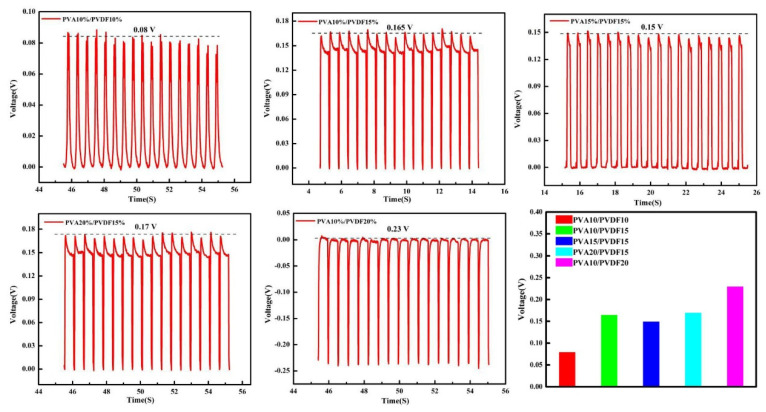
Piezoelectric responses of PVA/PVDF composite hydrogels.

The piezoelectric experimental results are shown in Figure 2. It can be seen that with the increase in the PVDF content in the hydrogel, the piezoelectric voltage of the materials increased from 0.08 V to 0.23 V. The reason for this result may be that under the same conditions, more PVDF in the hydrogel could be involved in the deformation, which would produce more dipole moments and cause the opposite charges at both ends of the material to increase [21,22]. In addition, it can also be seen that with the change in PVA content, the piezoelectric property of the material was changed too. When the content of PVA increased, the network structure of PVA became more compact and complete, and the PVDF attached to it also became more compact and complete, which resulted in the enhancement of piezoelectric voltage. However, with the increase in PVA content, the modulus of the material would also increase, causing the deformation of the material to become more difficult. Therefore, the obtained piezoelectric voltage of the material depends on a combination of the two factors. Generally speaking, the higher the PVDF content, the higher the piezoelectric voltage of the material.

Normally, we calculate the swelling rate by the change in mass, which is different from the swelling rate calculated by the change in volume. From the testing results of the swelling rate by the change of mass (Figure 1C), it can be seen that the swelling rate increased rapidly by 40–50% during the first one hour, and then showed a slow growth in the next two hours and finally reached equilibrium, achieving the highest swelling rate of about 60%. This was different from the changes in volume that we could see directly through macroscopic observation. The macroscopic results, as shown in Figure 1D, revealed that there was no significant volume change before and after swelling for materials. The reason for this difference is that the swelling rate calculated by the change of volume is different from the one calculated by the change of mass. The mass change-based swelling rate is associated with two different physical processes for porous materials. In the first stage, when water molecules enter the pores and occupy their position, this process does not result in the volume change of the system. At the second stage, when the pores of the system are completely occupied by water molecules, the curly polymer chains start to unfold under the action of water molecules; as a result, this leads to the increase of the volume. The first stage may be related to the capillary adsorption of water, while the second stage may be related to the hydrogen bond interaction between the hydroxyl group in the PVA chain and the water molecule [23]. From the swelling results, it is suggested that the swelling rate calculated by the change in mass for PVA is mainly contributed by the capillary adsorption of water. This is why we didn’t observe a significant change in PVA volume during swelling. This means that swelling rate does not play a dominant role in the screening of composites, so the focus of our attention would move to the piezoelectric effect.

Based on the above experimental results, PVA15/PVDF15 hydrogel with good comprehensive properties was selected for the follow-up experimental study. Furthermore, we hoped to endow PVA/PVDF hydrogels with antibacterial properties by adding Ag-NWs into the hydrogels and, meanwhile, to improve the piezoelectric property of the composites.

**Figure 3:**
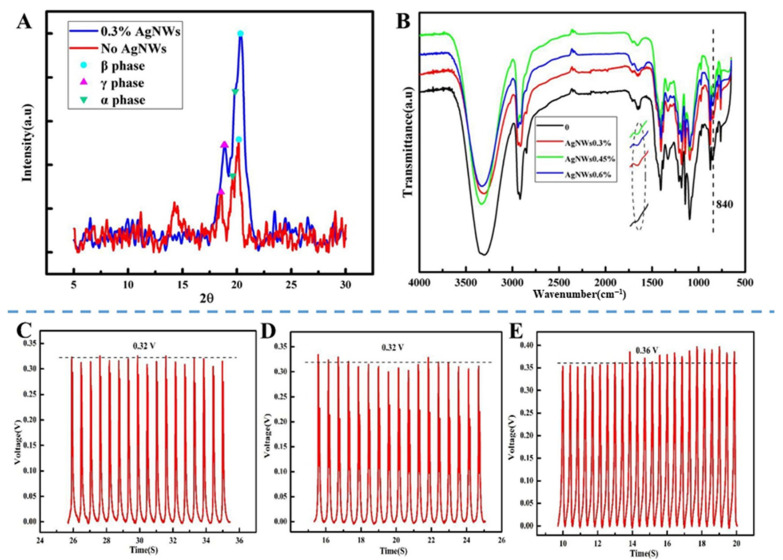
(**A**) XRD patterns of PVA/PVDF and PVA/PVDF/Ag hydrogels. (**B**) FT-IR spectra of PVA/PVDF and PVA/PVDF/Ag hydrogels. (**C**–**E**) Piezoelectric responses of PVA/PVDF hydrogels with 0.3% (**C**), 0.45% (**D**), and 0.6% (**E**) silver nanowires.

To study the effect of Ag-NWs on the piezoelectric property of materials, we first investigated the changes in XRD diffraction peaks before and after adding Ag-NWs. For PVDF, the characteristic peaks at 19.9°, 20.6° and 18.5° corresponded to α-phase, β-phase and γ-phase 19 [15], respectively. After the addition of Ag-NWs, the intensities of the peaks at 18.50° and 19.90° obviously decreased (as shown in Figure 3A). However, the characteristic peak of the piezoelectric β-phase at 20.6° was significantly enhanced compared with that before the addition of Ag-NWs, implying that the Ag-NWs contributed to the crystallization of β-phase in PVDF and improved the piezoelectric property of the material [24]. We further analyzed the hydrogel materials added with different proportions of silver nanowires by FT-IR, as shown in Figure 3B. There was no significant change in the spectra between the samples with and without Ag-NWs, indicating that Ag-NWs did not have any effect on the functional groups of PVA and PVDF. Comparing the characteristic peaks of β-phase at 840 cm^−1^ before and after the addition of Ag-NWs, it was found that the intensity of the peak at 840 cm^−1^ after adding different proportions of Ag-NWs had been enforced. These results demonstrate that Ag-NWs are beneficial for the formation of β-phase. However, it is difficult to quantitatively determine the relative content of the β-phase by using the traditional integral area method because PVDF is a semi-crystalline polymer with a complex structure. For that, we used Lambert Beer Law to perform a semi-quantitative analysis of the relative content of β-phase F(β) in PVDF. Formula (1) is as follows [25]:

(1) $$F\left(\beta \right)=\frac{A_{\beta }}{\left(\frac{K_{\beta }}{K_{\alpha }}\right)A_{\alpha }{+A}_{\beta }}$$

In the formula, Aα and Aβ are the absorbance at 766 cm^−1^ and 840 cm^−1^, respectively, while Kα and Kβ are the absorption coefficients of α and β phases at the respective wavenumber, which are 6.1 × 10^4^ and 7.7 × 10^4^ cm^2^/mol, respectively [26]. The calculation results showed that F(β) was about 0.411 for those without the addition of Ag-NWs. When the added proportions of Ag-NWs were 0.3%, 0.45%, and 0.6%, respectively, F(β) was almost the same value of 0.440, showing a certain increase compared with that one before addition and revealing that the formation of the β-phase of PVDF could be induced by the addition of Ag-NWs, which was in good agreement with the XRD results. The enhancement of β-phase may be due to the strong electron-absorbing property of the Ag-NWs so that some charged fluorine atoms in PVDF are attracted onto the surface of the Ag-NWs. Moreover, due to the linear structure of Ag-NWs, it is easy to force the fluorine atoms in the PVDF chains to gather on one side near each silver nanowire, while hydrogen atoms are distributed on the outer side of fluorine atoms, thus creating the planar trans (TTT) structure of β-phase, thus beneficial to the improvement of piezoelectric property [21,27].

Referring to the reported research results [22,27], in this study, we chose Ag doping amounts of 0.3%, 0.45%, and 0.6% to investigate the effect of Ag-NWs on piezoelectric properties. After adding Ag-NWs, the piezoelectric properties of the hydrogel are shown in Figure 3C–E. It can be seen that the piezoelectric voltage of the hydrogel increased from about 0.15 V to about 0.32 V, 0.32 V, and 0.36 V, respectively. However, an excessive amount of Ag-NWs will have a limited effect on the piezoelectric properties. Considering the biocompatibility of the material, we chose the Ag doping amount of 0.3% for the subsequent study.

### 3.2. Swelling Behaviors of Hydrogels

**Figure 4:**
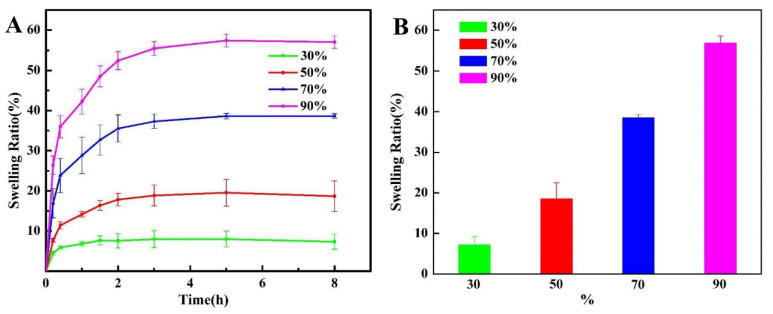
(**A**) Swelling curves and (**B**) swelling rates of the PVA/PVDF/Ag composite hydrogels dehydrated with different concentrations of ethanol in PBS buffer solution (37 °C, pH = 7.4).

In order to further allow the composite to better match the cartilage modulus, we used different concentrations of ethanol to dehydrate the composite, adjusted its compression modulus, removed the residual dimethyl sulfoxide, and then characterized the swelling property of the dehydrated sample, as shown in Figure 4. It can be seen that the material reached a swelling equilibrium state after 8 h. With the increase in ethanol concentration, the mass swelling rate of the hydrogel increased from nearly 10% to about 60%, and the swelling rate and ethanol concentration were positively correlated. However, the overall swelling rate of the hydrogel was not too high, and the maximum swelling rate was only 60%, which was similar to that of the previous material. The volume swelling rate of the material observed by the naked eye has no obvious change, further indicating that the swelling property of the material is suitable for osteochondral defect implantation.

### 3.3. Rheological Studies of Hydrogels

**Figure 5:**
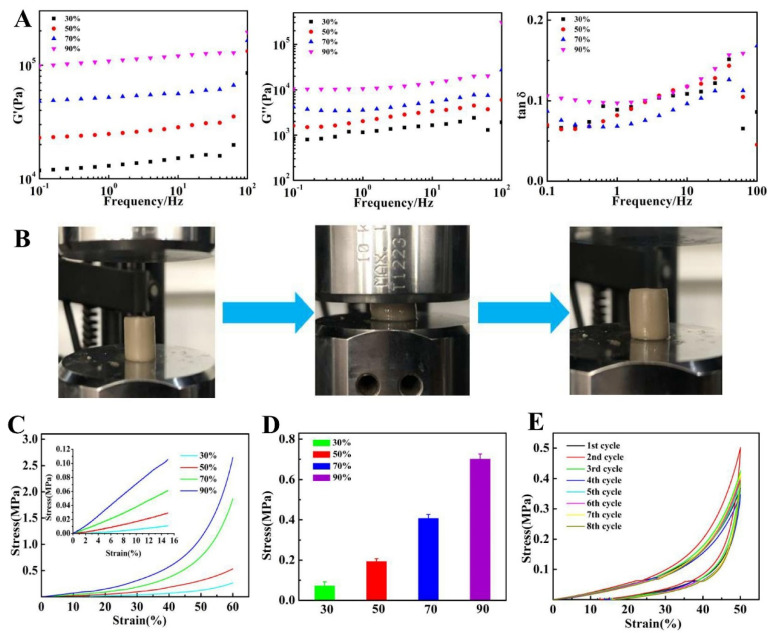
Dynamic rheology behaviors and mechanical properties of the PVA/PVDF/Ag composite hydrogels. (**A**) G’, G” and tan *δ* change with frequency. T = 25 °C, strain = 1%. (**B**) Compression process images of PVA/PVDF/Ag composite hydrogels. (**C**) Stress-strain curves of PVA/PVDF/Ag composite hydrogels under compression. (**D**) Compression modulus of PVA/PVDF/Ag composite hydrogels. (**E**) Cyclic compression stress-strain curve of PVA/PVDF/Ag composite hydrogels after 9 cycles.

The dynamic mechanical properties of PVA15/PVDF15/Ag hydrogels after dehydration using different ethanol concentrations are shown in Figure 5A. In the range of 0.1 to 100 Hz, the hydrogel had a high storage modulus (G′) and loss modulus (G″), which indicated that there were abundant crosslinking networks in the hydrogels so that it could withstand a large mechanical strength. The loss factor (tan δ) can reflect the energy loss caused by the relaxation or dissipation of viscoelastic materials [28,29]. With the increase in ethanol concentration, both storage and loss moduli of hydrogels increased, but the loss factor tan δ of the material had no significant change. This is because the volume of the material shrinks after the dehydration of ethanol, and the PVA network structure in the hydrogel is more closely combined with PVDF, so that the material can withstand higher mechanical strength under dynamic stress. Meanwhile, the volume shrinkage of the hydrogel makes the relative movement of the molecular chain more difficult and thus increases friction loss. As a result, the loss factor tan δ of the material remains unchanged.

### 3.4. Mechanical Properties of Hydrogels

The compressive property of the materials dehydrated with different ethanol concentrations is shown in Figure 5B–D, which shows typical nonlinear viscoelastic behavior. Therefore, the compressive modulus was measured on the basis of the slope of the linear portion of the stress-strain curve (strain 0–15%). It can be seen that with the increase in ethanol concentration, the compressive modulus of the hydrogel increased from 0.074 MPa to 0.703 MPa, which reached the level of 0.31 MPa of natural hyaline cartilage [30]. The PVA network structure formed by hydrogen bond cross-linking plays an important role in compressive resistance [31]. At the same time, the movement of the PVA chain was also restricted by PVDF that attached to the PVA chain and filled in the pore; their synergistic effect promoted the compressive strength of the hydrogel. It can also be seen that with the increase in the concentration of ethanol, the compressive modulus of the hydrogel increased [32]. 

The compression and recovery properties of hydrogel, which were closest to the natural modulus of cartilage. The results showed that after 9 cycles, the compression modulus of the hydrogel was still suitable for natural articular cartilage and showed good compression recovery performance (Figure 5E). When the strain reaches 50%, the hysteresis loop of the material appears to exhibit a weak hysteresis phenomenon, indicating that the material has weak energy dissipation. This is due to the dipole-dipole interaction mechanism in the PVDF molecular chain, which can heal the damage at the deformation point.

### 3.5. Morphology Analysis

**Figure 6:**
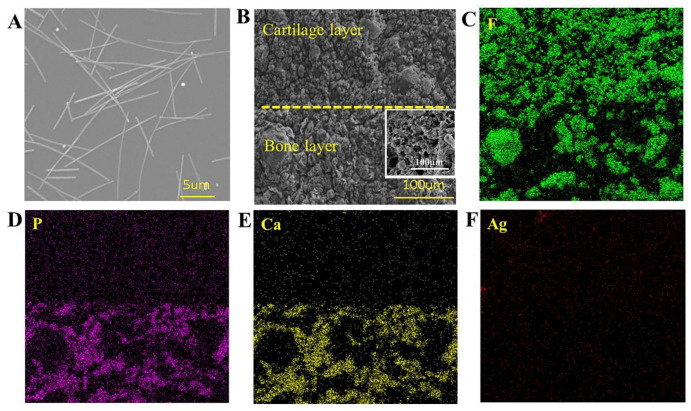
(**A**) SEM images of silver nanowires. (**B**) SEM images of PVA/PVDF/Ag composite hydrogels. (**C**–**F**) EDS mapping images of double-layer hydrogel. (**C**–**F**) have the same scale as (**B**).

Figure 6A,B shows the SEM images of silver nanowires and PVA15%/PVDF15%/Ag hydrogels. It can be seen that the length of the Ag-NWs obtained was about 5–15 μm. At the same time, the as-obtained hydrogel had a three-dimensional network structure with a pore size range of 10 to 50 μm, and both PVA and PVDF presented a two-phase separation structure, which is consistent with the previous characterization results. The EDS images showed that PVDF and Ag-NWs were evenly distributed in the hydrogel, as shown in Figure 6C–F, which was beneficial to PVDF β-phase formation and antibacterial property, while nano-HA was mainly concentrated in the bone layer, as was characterized by EDS images of elements P and Ca.

### 3.6. Cell Compatibility of Hydrogels

**Figure 7:**
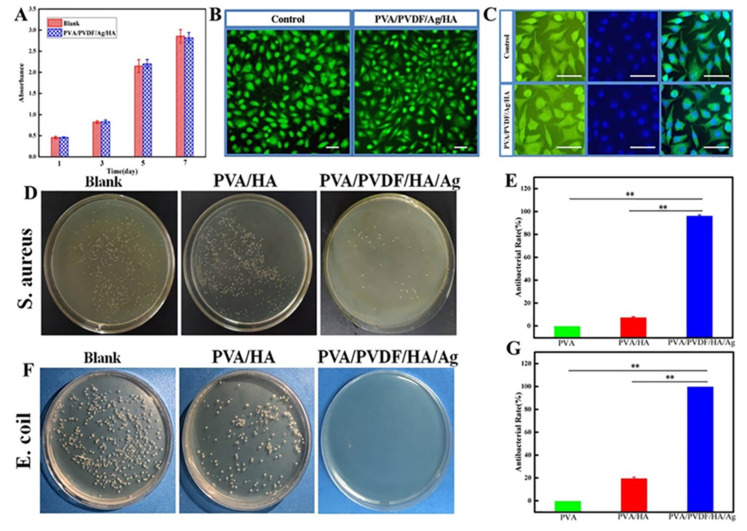
Cytotoxicity and antibacterial properties of PVA/PVDF/HA/Ag composite hydrogels. (**A**) Proliferative activity of L929 cells co-cultured with PVA/PVDF/HA/Ag composite hydrogels at 1 day, 3 days, 5 days, and 7 days. (**B**) Live/dead staining of L929 cells co-cultured with hydrogel after 7 days (all live nucleated cells generate a green fluorescence and all dead nucleated cells generate red fluorescence). Scale bars = 200 μm. (**C**) Fluorescence images of the L929 cells after 7 days of culture under F-actin/DAPI staining. Scale bars = 100 μm. (**D**) Images of the co-culture of *S. aureus* and composite hydrogels after 24 hours. (**E**) Antibacterial rate after co-culture of *S. aureus* with composite hydrogels after 24 h. (**F**) Images of co-culture of *E. coli* and composite hydrogels after 24 h. (**G**) Antibacterial rate after co-culture of *E. coli* with composite hydrogels after 24 h (** *p* < 0.01).

Based on the results of compression, piezoelectric and swelling properties of piezoelectric composite hydrogels, we finally chose 70% PVA15/PVDF15/Ag0.3 dehydrated composite hydrogel as the experimental group for subsequent biological evaluation, because its comprehensive properties are best matched with natural cartilage. We then studied the cell compatibility of piezoelectric hydrogel by means of co-culture of materials and L929 cells, and determined the cell viability at days 1, 3, 5, and 7 with the MTT method. Then, the cells on the 7th day were stained with live/dead cell staining, and the results are shown in Figure 7A,B. It can be seen that the cells in the blank group and the experimental group presented the same growth trend, and the absorbance of both groups showed no obvious difference. No death cells (which can be stained in red) were observed (Figure 7B). The F-actin/DAPI staining results showed that the morphology and structure in the experimental group were not significantly different from those in the blank group, and all the cells were in good condition, and the cell structure pseudopodia could be seen, which indicates that the piezoelectric hydrogel had good biocompatibility and thus was suitable for repairing the cartilage defects (Figure 7C). 

### 3.7. Antibacterial Properties of Hydrogels

Further, we evaluated the antibacterial properties of piezoelectric composite hydrogels at the macroscopic level by using the plate counting method. Because the plate counting method is better than the bacteriostatic ring method, it is beneficial to evaluate the bacteriostatic effect of the release of silver ions in materials. Figure 7D–G shows the results of the in vitro antibacterial experiment. It can be seen that a large number of colonies were formed on the Petri dishes after the co-culture of bacteria with the blank group and PVA/HA hydrogel group for 24 h. In sharp contrast, the culture dish that was co-cultured with the piezoelectric composite hydrogel was basically sterile, and the results were the same for the culture of both Escherichia coli and Staphylococcus aureus. Through quantitative calculation, the inhibition rates of piezoelectric hydrogel on two kinds of bacteria reached over 95%, indicating that the piezoelectric composite hydrogel had good antibacterial properties. We believe that the main antibacterial mechanism should be attributed to the release of ions from Ag-NWs. Positively charged Ag ions can be adsorbed on the surface of the bacterial cell membrane by electrostatic interaction and react with the cell membrane to produce peptidoglycan, which affects the permeability of the bacterial membrane and destroys the electron and matter transport systems of microorganisms. Furthermore, Ag ions penetrate the cell wall, leading to cell wall rupture and cytoplasm outflow. At the same time, they can also bind with some groups in the bacteria, such as -SH, to coagulate the protein and destroy the activity of synthetase, thus killing the bacteria [33,34].

### 3.8. Hemocompatibility Assay

**Figure 8:**
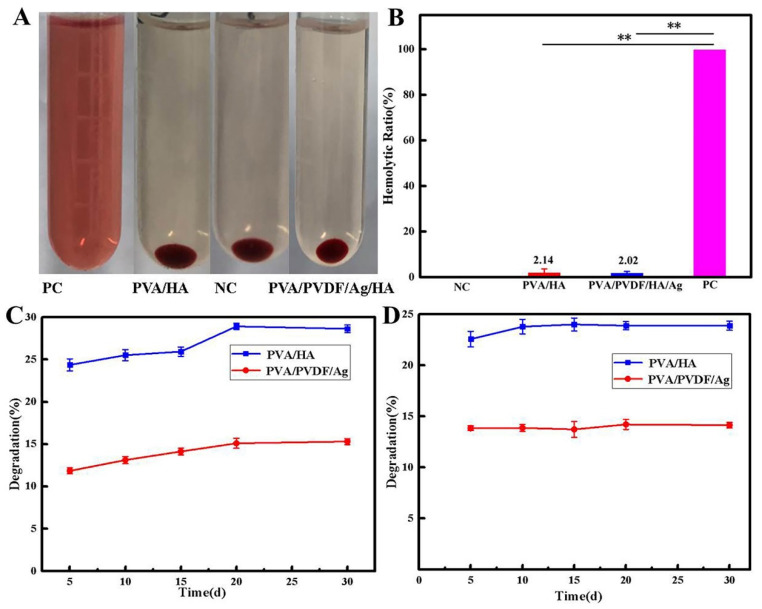
(**A**) Photos of the hemolysis assay of composite hydrogels. (**B**) Hemolysis rate of the composite hydrogels. (**C**,**D**) Degradation rate of composite hydrogels in PBS buffer solution (pH = 7.4) and in PBS buffer solutions with trypsin, respectively (** *p* < 0.01).

We further used hemolysis to determine the blood compatibility of PVA/HA hydrogel and PVA15/PVDF15/Ag0.3 hydrogel, and the results are shown in Figure 8A,B. It can be seen that both hydrogels had almost no damage to red blood cells, and no obvious hemolysis occurred compared with the positive control group. The hemolysis rates of both PVA/HA and PVA15/PVDF15/Ag0.3 hydrogels were lower than 5% in the quantification test of the hemolysis rate (Figure 8B), according to the application standard of biomaterials.

Figure 8C,D shows the results of in vitro degradation of hydrogel materials. It can be seen that the material basically did not degrade very well in the environment of both PBS buffer (pH = 7.4) and trypsin solutions. Generally speaking, the degradation principle of polymer materials is that water molecules and enzymes cut off the polymer molecular chain, leading to the degradation of the materials [35,36]. In this experiment, the degradation rate of PVA remained at 25%, and the degradation rate of the material decreased after adding PVDF, which can be attributed to the hydrophobic effect of PVDF, resulting in the decreased effect of water molecules on the polymer chain [37]. The lower degradation rate of the material can provide better support for us to verify the mechanism of piezoelectric-enhanced osteochondral repair.

### 3.9. General Observation and Histological Analysis of Osteochondral Repair

**Figure 9:**
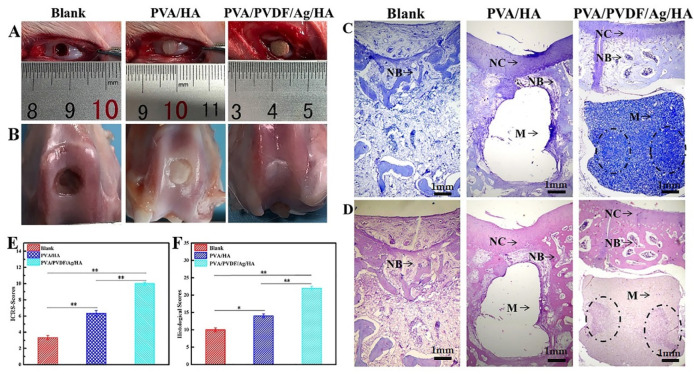
(**A**) Osteochondral defect model in rabbits. (**B**) Macroscopic images of knees repaired after 12 weeks (**C**,**D**).Histological analysis of the repaired knees after 12 weeks. (**C**) Toluidine blue staining. (**D**) Hematoxylin and eosin staining. (**E**) (International Cartilage Repair Society) scores of blank group, PVA/HA group, and PVA/PVDF/Ag/HA group at 12 weeks post-surgery. (**F**) Histological scoring for evaluating osteochondral defects (* *p* < 0.05, ** *p* < 0.01). NC: new cartilage; NB: new bone; M: scaffold materials. The area marked with circles showed the ingrowth of new bone.

It can be seen from Figure 9A,B that there is still obvious depression in the defect of blank group, and there is basically no repair effect. In the PVA/HA hydrogel group, there was a clear difference between the defect and the surrounding tissues, and their colors were quite different. Only a small amount of cartilaginous tissue was formed, which was not significantly different from that of the pure PVA hydrogel previously reported [38]. In the piezoelectric hydrogel group, there were no obvious sunken surfaces in the defect area, the boundaries disappeared, and the surrounding tissues were well combined with the newly formed tissues. It is difficult to tell the color difference between new tissue and host tissue. The results show that the piezoelectric effect can enhance tissue repair. It is generally believed that when the piezoelectric scaffold is implanted into the damaged cartilage, it can deform under the action of mechanical stress, resulting in an electrical potential difference, which, as an electrical signal, will then regulate the Ca^2^+ channel and promote the influx of calcium [39,40]. A further increase of intracellular Ca^2^+ concentration will activate calmodulin and dephosphorylate the nuclear factor of activated T cells (NF-AT) located in the nucleus (activated calcineurin). Ultimately, these will lead to the expression of genes responsible for growth factors, such as transforming growth factor TGF-β and BMP, thus promoting the regeneration of cartilage tissue [41,42].

Further, we evaluated the formation of osteochondral tissues as well as the integration of materials and host tissues at the defects by H&E and toluidine blue staining of histological sections at the defects at 12 weeks after operation, as shown in Figure 9C,D. No chondrocyte formation was observed in the defect area of the blank group at 12 weeks after the operation. Although there were a few newly formed subchondral bone and cartilage tissues in the PVA/HA group, the degree of chimerism between chondrocytes and the subchondral bone was not obvious. The cartilage and subchondral bone of the defects in the PVA/PVDF/Ag/HA group were repaired, the physiological structure of the cartilage was normal and complete, the interface was closely bound, and there was no obvious boundary between NC (new cartilage) and NB (new bone). These results indicate that piezoelectric hydrogel effectively promoted the repair of osteochondral defects. Furthermore, it can be seen that no tissue grew into the scaffold, and that the bonding between the scaffold materials and tissues in the PVA/HA group was so poor that the scaffold materials came off the histological section. While the bonding between the scaffold materials and tissues in the piezoelectric group was strong, it can be seen that new bone tissue has grown into the subchondral bone layer of the scaffold. The ICRS scores (Table 1) from macroscopic observation illustrated that the average scores reached 10 in the PVA/PVDF/Ag/HA group, which was significantly higher than that of the blank and PVA/HA groups at 12 weeks post-implantation (Figure 9E). The results from the histological scoring system (Table 2) showed that the average score of the piezoelectric hydrogel for cartilage repair was 22, significantly better than that in both the blank and PVA/HA groups (Figure 9F).

In addition, we measured the size of the implants and found that the height of the implants at 12 weeks after implantation was generally less than that before implantation. We speculated that the reduction in implant size was most likely due to the degradation of the implants in vivo, showing that PVA has a certain biodegradation ability in vivo. In contrast, PVA showed a higher biodegradation rate in vivo than the PVA/PVDF composite. However, it is interesting to see that the degradation area for the PVA/PVDF composite mainly occurred at the top part of the scaffold, namely, the main stress area of the scaffold materials, which suggests that the piezoelectric effect might improve the degradation ability of the implants by means of activating the activity of enzymes or other degradation mechanisms.

In general, the piezoelectric-effect-enhanced tissue regeneration ability has been seen, and the piezoelectric-effect-enhanced degradation was also observed in our present PVA material system, showing potential values for future application in bionics principle-and piezoelectric effect-based osteochondral tissue engineering.

## 4. Conclusions

In this study, a composite hydrogel with piezoelectric and antibacterial properties was prepared, and these properties were enhanced by introducing silver nanowires. The mechanical and swelling properties of the material have been successfully tuned to achieve a compression modulus similar to that of natural articular cartilage and a low swelling rate that could meet the bone and cartilage tissue engineering application. Based on these and the bionics principle, a double-layer piezoelectric hydrogel was successfully prepared for osteochondral regeneration and has shown an enhanced repair effect of osteochondral defects compared with the hydrogel without the piezoelectric phase. Meanwhile, the introduction of the piezoelectric phase can also stimulate the in vivo degradation of the materials by means of activating the activity of enzymes or other degradation mechanisms. These results have shown that piezoelectric effect-based composite materials have good application prospects in tissue engineering.

## Figures and Tables

**Table 1:** Cartilage Repair Assessment ICRS.

Scoring Items	Index Parameters	Score
Degree of Defect Repair	Complete repair of defect depth	4
	75% repair of defect depth	3
	50% repair of defect depth	2
	25% repair of defect depth	1
	0% repair of defect depth	0
Integration to Border Zone	Complete integration with surrounding cartilage	4
	Demarcating border <1 mm	3
	3/4th of graft integrated, 1/4th with a notable border >1 mm width	2
	1/2 of graft integrated with surrounding cartilage,1/2 with a notable border >1 mm	1
	Form no contact to 1/4th of graft integrated with surrounding cartilage	0
Macroscopic Appearance	Intact smooth surface	4
	Fibrillated surface	3
	Small, scattered fissures or cracks	2
	Several, small or few but large fissures	1
	Total degeneration of grafted area	0
Overall Repair Assessment	Grade I: normal	12
	Grade II: nearly normal	11–8
	Grade III: abnormal	7–4
	Grade IV: severely abnormal	3–1

**Table 2:** Histological scoring system for evaluating osteochondral defects.

Scoring Items	Index Parameters	Score
(a) Overall Defects Evaluation		
1. Percent Filling With Newly Formed Tissue	100%	3
	>50%	2
	<50%	1
	0	0
2. Percent Degradation of the Implant	100%	3
	>50%	2
	<50%	1
	0	0
(b) Subchondral Bone Evaluation		
3. Percent Filling with Newly Formed Tissue	100%	3
	>50%	2
	<50%	1
	0	0
4. Subchondral Bone Morphology	Normal, trabecular bone	4
	Trabecular bone, with some compact bone	3
	Compact bone	2
	Compact bone and fibrous tissue	1
	Only fibrous tissue or no tissue	0
5. Extent of New Tissue Bonding with Adjacent Bone	Complete on both edges	3
	Complete on one edge	2
	Partial on both edges	1
	Without continuity on either edge	0
(c) Cartilage Evaluation		
6. Morphology of Newly Surface Tissue	Exclusively articular cartilage	4
	Mainly hyaline cartilage	3
	Fibrocartilage	2
	Only fibrous tissue	1
	No tissue	0
7. Thickness of New Formed Cartilage	Similar to surrounding cartilage	3
	Greater than the surrounding cartilage	2
	Less than the surrounding cartilage	1
	No cartilage	0
8. Joint Surface Regularity	Smooth, intact surface	3
	Surface fissures	2
	Deep fissures	1
	Complete disruption of the new surface	0
9. Chondrocyte Clustering	None at all	3
	<25%	2
	25~100%	1
	No chondrocytes present	0

## Data Availability

Not applicable.
